# Impact of comorbidities on gout and hyperuricaemia: an update on prevalence and treatment options

**DOI:** 10.1186/s12916-017-0890-9

**Published:** 2017-07-03

**Authors:** Thomas Bardin, Pascal Richette

**Affiliations:** 1 Université Paris Diderot, UFR médicale, Paris, France; 2 Assistance Publique-Hôpitaux de Paris, Hôpital Lariboisière, Service de Rhumatologie, Paris, Cedex France; 3 INSERM 1132, Université Paris-Diderot, Hôpital Lariboisière, Paris, France; 4 French-Vietnamese Research Center on Gout, Ho Chi Minh City, Vietnam

**Keywords:** Obesity, Hypertension, Type-2 diabetes, Dyslipidaemia, Coronary heart disease, Heart failure, Atrial fibrillation, Cardiovascular death, Colchicine, Urate-lowering drugs

## Abstract

Gout, the most prevalent inflammatory arthritis worldwide, is associated with cardiovascular and renal diseases, and is an independent predictor of premature death. The frequencies of obesity, chronic kidney disease (CKD), hypertension, type 2 diabetes, dyslipidaemias, cardiac diseases (including coronary heart disease, heart failure and atrial fibrillation), stroke and peripheral arterial disease have been repeatedly shown to be increased in gout. Therefore, the screening and care of these comorbidities as well as of cardiovascular risk factors are of outmost importance in patients with gout. Comorbidities, especially CKD, and drugs prescribed for their treatment, also impact gout management. Numerous epidemiological studies have shown the association of asymptomatic hyperuricaemia with the above-mentioned diseases and cardiovascular risk factors. Animal studies have also produced a mechanistic approach to the vascular toxicity of soluble urate. However, causality remains uncertain because confounders, reverse causality or common etiological factors might explain the epidemiological results. Additionally, these uncertainties remain unsolved despite recent studies using Mendelian randomisation or therapeutic approaches. Thus, large randomised placebo-controlled trials are still needed to assess the benefits of treating asymptomatic hyperuricaemia.

## Background

Gout is a prevalent disorder whose frequency is increasing worldwide [1]. In addition to causing excruciating arthritic pain, gout is associated with premature death [2], classically explained by a high frequency of comorbidities, especially renal and cardiovascular diseases [3]. Comorbidities must be considered in gout because they could contribute to the vital prognosis of gouty patients and they complicate gout management. These comorbidities are also frequently associated with asymptomatic hyperuricaemia, an even more prevalent condition than gout [4], with their causal relationship with hyperuricaemia raising important therapeutic issues.

## Cardiovascular and renal comorbidities in gout

The association of gout with cardiovascular and renal diseases, observed as early as the late nineteenth century [5], is now well established. The prevalence of comorbidities increases with gout duration [6] and gout is associated with all components of the metabolic syndrome [7]. Hypertension is frequent; in the third National Health and Nutrition Examination Survey (NHANES), the prevalence of hypertension was 69.1% (95% CI, 59.4–78.8) and 30.3% (95% CI, 28.4–32.2) in gouty and non-gouty patients, respectively [7]. Prospective studies have consistently shown increased risk of gout in hypertensive patients [8, 9], and in the recent Singapore Chinese Health Study, the risk of hypertension was increased for gouty patients [10].

In the third NHANES, the prevalence of abdominal obesity was greater for gouty than non-gouty patients, at 62.9% (95% CI, 50.9–74.8) versus 35.3% (95% CI, 33.7–36.9) [7]. Additionally, risk of gout increases with obesity [8]. Body mass index (BMI) and waist circumference are correlated with uricaemia [11], and adiposity was recently found to be a cause of hyperuricaemia [11].

Type 2 diabetes mellitus prevalence is increased in gouty patients; in the third NHANES, diabetes prevalence was greater for gouty than non-gouty patients, at 33.1% (95% CI, 28.8–41.4) versus 10.8% (95% CI, 9.9–11.8). In prospective studies, gout also increased the risk of type 2 diabetes mellitus [12, 13], whereas diabetes lowered the risk of incident gout [13]; the latter finding was explained by the fact that glycosuria increases urine excretion of urate [14]. The lower incidence of gout in type 2 diabetes might also partly be explained by the frequent prescription of metformin, which may lead to an anti-inflammatory effect through modulation of different cellular pathways, including AMP-activated protein kinase, protein kinase A and PPAR gamma [15, 16].

Hypertriglyceridaemia prevalence was found to be greater in gouty patients than non-gouty individuals, at 53.7% (95% CI, 42.9–64.4) versus 27.9% (95% CI, 25.5–30.3) [7], and a recent Mendelian randomisation study found that it caused hyperuricaemia [17]. The frequency of an elevated low-density lipoprotein cholesterol level was higher in gouty patients than non-gouty individuals in the third NHANES, at 47.4% (95% CI, 37.2–57.6) versus 36.6% (95% CI, 34.1–39.1).

The prevalence of chronic kidney disease (CKD) of stage 3 or above in gout was estimated at 24% (95% CI, 15–28) in a recent meta-analysis of six studies [18]. Reduced kidney function decreases urate excretion in urine and increases the risk of gout. In a large German CKD patient cohort [19], gout prevalence was found to increase from 16.0% to 35.6% for CKD patients with an estimated glomerular filtration rate above 60 mL/min/1.73 m^2^ versus those with a rate below 30 mL/min/1.73 m^2^. Conversely, the risk of end-stage renal failure was found to be increased in patients with gout [20]. This finding can be explained by the formation of uric acid crystals in the renal tubules [21], interstitial nephritis complicating kidney stones, crystalline deposits in the renal medulla [22], use of non-steroidal anti-inflammatory drugs (NSAIDs), a frequent association with hypertension, or the possible renal toxicity of soluble uric acid [23].

Various cardiac diseases are independently associated with gout [24] and thereby associated with increased frequency of cardiovascular deaths [25–27]. In a large cohort of gout patients, cardiovascular diseases accounted for more than half of the deaths, and cardiovascular mortality increased with gout severity [27]. Hyperuricaemia might partially account of the increased cardiovascular risk of gouty patients, as discussed below. However, multivariate-adjusted cardiovascular risk appears to be more important and less disputable in gout than in asymptomatic hyperuricaemic patients, suggesting that crystal-driven inflammation plays an important role [28], in line with the increased risk observed in rheumatoid arthritis, psoriatic arthritis or ankylosing spondyloarthritis. Adjusted risk of coronary heart disease is increased for both men [26, 29] and women [30] with gout. Heart failure is associated with gout [31] and hyperuricaemia, which is worsened by the use of diuretics and is a marker for poor outcome [32]. The risk of atrial fibrillation is increased in gout and the prevalence of atrial fibrillation at gout diagnosis was recently estimated to be 7.4% (95% CI, 7.2–7.6) in a large UK database [33]. Gout has also been recently found to be associated with aortic stenosis [34]. Additionally, gout was recently confirmed to increase the adjusted risk of ischemic stroke and peripheral vascular disease [24]. Our cluster analysis of gouty patients revealed that obesity is preferentially associated with hypertension, type 2 diabetes mellitus and dyslipidaemia, whereas cardiovascular and CKD are frequent in patients with a high rate of diuretic prescriptions [35].

## Hyperuricaemia and cardiovascular and renal diseases

Over the last 15 years, the association of hyperuricaemia with cardiovascular diseases has been re-examined following the demonstration in animal models that hyperuricaemia could cause vascular disease. In rodents, hyperuricaemia induced by inhibiting uricase [36], enriching the diet with fructose [37], or deleting the intestine glucose transporter 9 (GLUT9) urate transporter [38] led to features of metabolic syndrome and renal atherosclerosis. Many of the induced features, especially hypertension, were prevented by the early administration of urate-lowering drugs (ULDs). In addition, hyperuricaemia activated the renin-angiotensin system, decreased nitric oxide synthase activity, stimulated vascular smooth muscle cell proliferation, and promoted insulin resistance. These features provided a mechanistic model showing how soluble intracellular uric acid could damage the endothelium and arteries, in contrast to its classical antioxidant properties now believed to be limited to an extracellular effect [5]. Moreover, in humans, hyperuricaemia was recently found to be associated with increased lipid levels and decreased fibrous volume, potentially leading to increased plaque fragility [39, 40], representing a possibly important link to arterial obstruction. Another human study found birefringent crystals in six alcohol-fixed coronary arteries from 55 explanted hearts, suggesting that monosodium urate (MSU) crystal inflammation could occur in coronary arteries [41].

A large number of epidemiological studies have explored the link between hyperuricaemia and cardiovascular and renal outcomes [28, 42], and suggested an association of hyperuricaemia with increased frequency of cardiovascular death [43], coronary heart disease [44], heart failure, atrial fibrillation [45] and stroke [46]. Recently, a cross-sectional study suggested that coronary heart disease could be more severe in hyperuricaemic patients with asymptomatic MSU crystal deposition than normouricaemic or hyperuricaemic patients with no crystal deposits [47]. Prospective cohorts have shown that hyperuricaemia leads to hypertension [48], which was recently found to be more refractory when associated with hyperuricaemia [49, 50], renal failure [23], type 2 diabetes mellitus [51] and metabolic syndrome [52]. However, these impressive results did not prove causality because the observed associations could be explained by confounding factors, reverse causality or the intervention of a common causal factor. Since hyperuricaemia is associated with numerous cardiovascular risk factors, confounders could fully account for an adverse outcome, as observed in the Framingham Heart Study [3]. Most of the later studies have observed an association between hyperuricaemia and poor cardiovascular outcomes, despite multiple adjustments for cardiovascular risk factors [43]. However, adjustments were frequently incomplete, with some studies not adjusting for BMI, smoking, insulin resistance and renal function. Additionally, dietary factors associated with hyperuricaemia and possibly increased risk of death (i.e. alcohol consumption) were not taken into account. Despite the temporal relationship (hyperuricaemia was found to precede hypertension, CKD and metabolic syndrome), reverse causality cannot be excluded. Indeed, slight renal dysfunction or hyperinsulinemia, initially not revealed, could explain hyperuricaemia. As an example, familial juvenile hyperuricaemic nephropathies were first recognised in young patients with early gout/hyperuricaemia and preceded severe renal failure. Early treatment with allopurinol was observed to protect some patients from the renal failure observed in the disease, which led to the hypothesis that hyperuricaemia was the cause of renal dysfunction. Genetic studies have now established that the disease has a renal origin, due to a number of genetic variants of the uromodulin or hepatocyte-nuclear factor 1 b genes [53]. The observed influence of allopurinol now appears to be a non-specific renoprotective effect, observed in various nephropathies (see below).

Persisting uncertainties regarding the causal relationship between hyperuricaemia and cardiovascular disease has recently led a number of investigators to use Mendelian randomisation to investigate the effect of urate-governing genetic variants on various cardiovascular outcomes [11]. Mendelian randomisation allows a comparison of cardiovascular features in patients with and without hyperuricaemic genes. Because these genes are randomly distributed during meiosis, confounding factors should be equilibrated between study participants with and without the hyperuricaemic variants as they are in a randomised clinical trial, thus avoiding the use of adjustments and their uncertainties. In addition, because genetic variants are not affected by health outcomes, associations are not affected by reverse causality [54]. Although heritability appears stronger for uricaemia than gout [55], genetic factors governing uricaemia are many [56], still partly unknown, and explain only a small fraction of the uricaemia variance, especially when hyperuricaemic variants are considered individually, which leads to the risk of underpowered studies. Variants of the solute carrier family 2 member 9 (*SLC2A9*) gene, which encodes for the GLUT9 transporter and accounts for 3.5% of the uricaemia variance in males and up to 15% in females [57], were used as an instrumental variable in most of the first studies (Table 1). Studies of relatively small samples of Amish people and from southern Italy, which are relatively homogeneous populations, supported a causal role of hyperuricaemia in the development of hypertension [58, 59], atherosclerosis [60] or renal failure [61]. However, a study of two large Danish cohorts did not support causality for coronary heart disease or hypertension [62], and a further study did not find evidence between hyperuricemia and the metabolic syndrome [63].

**Table 1:** Mendelian randomisation studies of the link between uricaemia and various cardiovascular diseases/risk factors with variants of *SLCA9* as instrumental variables

Author year [ref]	Instrumental variable	No. of participants	Population	Main results
McKeigues 2009 [63]	SNP	1017	Orkney Islands Scottish	No effect on metabolic syndrome
Parsa 2012 [58]	SNP	516	Amish	Increase in ambulatory SBP, with low- and high-salt diets
Palmer 2013 [62]	SNP	>58,000 >10,000	Danes	No effect on BP or ischemic heart disease
Testa 2014 [61]	SNP	755	Southern Italy	Prediction of CKD progression
Mallamaci 2015 [59, 60]	SNP	449	Southern Italy	Increase in office SBP and US carotid atherosclerosis features

*BP* blood pressure, *SBP* systolic blood pressure, *SNP* single nucleotide polymorphism, *CKD* chronic kidney disease, *US* ultrasonography

Genetic risk scores (GRSs), which could be weighted to account for the effect of each gene on uricaemia, were used in other studies in an attempt to increase the genetic impact on uricaemia (Tables 2 and 3). Two studies supported causality for cardiovascular death, sudden cardiac death [64] and diabetic macro-angiopathy [65]. However, many studies [17, 66–72], even when largely powered, reached opposite conclusions, despite two of them showing an association of GRSs with gout [66, 71] (Table 3). The Rotterdam cohort showed a negative association between hyperuricaemic GRS and blood pressure, even stronger in patients receiving diuretics [69]. The use of several gene variants in the GRS increased the possibility that some of the included genes (or genes in linkage disequilibrium with them, which are not independently distributed during meiosis) had other effects than their soluble uric acid (SUA)-modulating property, a phenomenon called pleiotropy, which could be an important confounder. Pleiotropy was thought to explain that a hyperuricaemic GRS, consisting of five renal transporter gene variants, was found to be associated with the preservation of renal function and not with its worsening, as was observed in many epidemiologic studies [68]. Recent studies have attempted to account for pleiotropy, including the exclusion of genes that appeared as pleiotropic by analysing their effects on biological (i.e. lipids) or clinical parameters (i.e. blood pressure, BMI) in the studied cohorts [65, 71], adjustment for confounders [72], or using Egger randomisation, which accounts for hidden pleiotropy [72]. Thus, despite some discrepant results [64, 65], Mendelian randomisation studies do not appear to support a causal link between SUA level and cardiovascular outcomes (Table 3) [73, 74].

**Table 2:** Mendelian randomisation studies using genetic risk scores (GRSs) to explore causality between uricaemia and cardiovascular outcomes; positive studies

Author year [ref]	GRS instrumental variable	No. of participants	Population	Account of pleiotropy	Main results
Kleber 2015 [64]	8 SNPs	3315	LURIC cohort German	No pleiotropy found	Association with cardiovascular mortality and sudden cardiac death
Yan 2016 [65]	17 SNPs 14 excluded	T2DM >3200	China, Cross-sectional	Exclusion of 14 genes that are pleiotropic or in linkage disequilibrium	Association with diabetic macro-angiopathy

*GRS* genetic risk score, *SNP* single nucleotide polymorphisms, *T2DM* type 2 diabetes mellitus

**Table 3:** Mendelian randomisation studies using genetic risk scores (GRSs) to explore causality between uricaemia and cardiovascular outcomes; negative studies

Author year (ref)	Instrumental variable (GRS)	Effect on SUA variance	No. of participants	Population	Accounted for pleiotropy	Main results
Stark 2009 [73]	10 SNPs		CAD: 1473; controls: 1241	Germany	No	No association with CAD
Yang 2010 [66]	8 SNPs	6%	>50,000	Europe and United States	No	Association with gout but not with BP, glucose level, CKD, CAD
Pfister 2011 [67]	8 SNPs		T2DM: 7504; controls: 8560		No association of the GRS SNPs with confounders	No association with diabetes
Hughes 2013 [68]	5 SNPs, urate transporters	2%	>70,000	ARIC and FIJS cohorts	Likely to explain the observation	Association with improved renal function
Rasheed 2014 [17]	5 SNPs, urate transporters	2%	>70,000	ARIC and FIJS cohorts	No	No causal link with triglycerides; triglycerides cause hyperuricaemia
Sedaghat 2015 [69]	30 SNPs	2 mg/dL	5791	Rotterdam cohort	No	Negative association with SBP and DBP stronger in diuretic users
Sluijs 2015 [70]	24 loci	4%	>41,500	Europeans: EOIC interact and DIAGRAM cohorts	No pleiotropy except for triglycerides level	No association with diabetes
Yan 2016 [65]	17 SNPs 14 excluded		T2DM: >3200	China Cross-sectional	Exclusion of 14 pleiotropic genes or in linkage disequilibrium	Association with diabetic macro-angiopathy
Keenan 2016 [74]	28 SNPs 14 excluded		T2DM: 65,000; controls: 68,000 CHD: 54,500; controls: 68,275 HF: 4553; controls: 19,985 IS: 14,800; controls: 19,900	Europe South Asia	Exclusion of 14 pleiotropic genes	Association with gout No association with T2DM, CAD, stroke, HF
White 2016 [72]	31 SNPs		>166,000 >9800 CHD events	17 observational cohorts, mainly from the United Kingdom	Multivariate analysis Egger Mendelian randomisation	Multivariate OR 1–1.22; Egger OR 0.92–1.22

*BP* blood pressure, *CAD* cardiovascular disease, *CHD* cardiac heart disease, *DBP* diastolic blood pressure, *GRS* genetic risk score, *HF* heart failure, *OR* odds ratio, *SBP* systolic blood pressure, *SNP* single nucleotide polymorphism, *SUA* soluble uric acid IS ischemic stroke, *T2DM* type 2 diabetes mellitus

An alternative explanation for the observed associations between hyperuricaemia and cardiovascular disease could be the intervention of a common etiological factor. Xanthine oxidase (XO) is a candidate for such a role because it produces free oxygen species that inhibit the production of NO and may lead to endothelial damage [75, 76]. The hypothesis of increased XO activity in hyperuricaemic individuals remains unproven because of the difficulty in dosing the enzyme in living endothelial cells, and primary hyperuricaemia is believed to usually result from a lack of proper renal adaptation to SUA levels. However, hyperuricaemic dietary factors may also be involved in primary hyperuricaemia by increasing the enzyme activity, which is involved in the last two steps of uric acid production. Increased XO activity, rather than SUA, could be responsible for an adverse cardiovascular outcome. In line with this hypothesis, a recent study examined several XO gene variants in a prospective cohort of 2500 European participants and found three minor alleles associated with increased risk of hypertension [77]. Despite the enzymatic activity controlled by these variants being uncertain, another a longitudinal cohort study of 246 Dutch children (mean age 7 years) [78], led by the same investigator, showed that XO activity, estimated by a ratio of purine to uric acid, was associated with blood pressure. Therefore, increased XO activity, rather than SUA, could be responsible for the increased blood pressure frequently observed in hyperuricaemic individuals [79].

Causality can also be addressed by studying the effect of drugs. Most of the recent case–control retrospective studies [80–82], but not all [83], suggested that allopurinol, especially at > 300 mg/d, could improve the cardiovascular outcomes of patients with asymptomatic hyperuricaemia. However, these studies were subjected to potential bias because allopurinol may be a marker of better care and could be frequently associated with colchicine, which has been found to be effective for coronary heart disease [84]. Randomised controlled trials (RCTs) comparing allopurinol with placebo were recently reviewed [85], with most studies suggesting a beneficial effect on cardiovascular disease but including small numbers of patients. In contrast, a recent randomised placebo-controlled trial involving more than 250 patients failed to show that allopurinol (600 mg/d) mitigated hyperuricaemic heart failure [86]. Several small RCTs also suggested that allopurinol could slow the decline in renal function in hyperuricaemic CKD patients [87]. A recent monocentric RCT of 93 hyperuricaemic CKD patients suggested that febuxostat could have similar beneficial effects [88]. However, such a renoprotective effect of febuxostat was not observed in another recent RCT of gout patients with moderate-to-severe renal impairment [89].

The most convincing results come from two small RCTs in which allopurinol or probenecid corrected recent-onset hypertension in adolescents [90, 91]. However, these results cannot be extended to adults; a recent RCT reported that ULDs did not change blood pressure [92]. In rodents, uric acid-induced hypertension could be corrected by ULDs only in the early phase [93], and whether ULDs could correct hypertension in adults with long-lasting hyperuricaemia is uncertain. Results of large and well-designed RCTs are still awaited to assess the cardiovascular and renal benefits of treating asymptomatic hyperuricaemia.

## Impact of comorbidities on gout management

### Diagnosis and treatment of comorbidities

The high frequency of cardiovascular disease and the increased risk of cardiovascular death with gout led to the European League Against Rheumatism (EULAR) recommendation for systematic screening and care for patients with gout in terms of cardiovascular diseases and risk factors [94]. Even though smoking has been recently found to be negatively associated with gout [95–97], smoking cessation was included in the EULAR recommendations because of the increased cardiovascular risk associated with gout [94]. Weight control appears to be highly important as it can improve uricaemia and metabolic syndrome features.

Additionally, drugs targeting comorbidities may affect uricaemia. Cardioprotective aspirin increases uricaemia [98] and its onset has been associated with gout flares [99]. Antihypertensive drugs, such as beta-blockers, angiotensin converting-enzyme inhibitors, non-losartan angiotensin II receptor blockers, thiazide and loop diuretics, have been associated with increased risk of gout, and calcium channel blockers and losartan, which are uricosurics, should be preferred for gout [100]. Loop diuretics are often needed for patients with heart failure, but should be kept at the minimal effective dose. Spironolactone, with no effect on uricaemia [101], should be privileged. For the management of dyslipidaemia, fenofibrate [102] and atorvastatine [103] have urate-lowering effects. Similarly, for the management of type-2 diabetes mellitus, insulin-lowering drugs should be privileged because insulin decreases the urine output of urate. The recently introduced sodium-glucose co-transporter 2 inhibitors also have urate-lowering effects [104].

### Impact of comorbidity on management of gout inflammation

Comorbidities can imply contraindication for drugs usually prescribed for the management of acute flares in gout [105]. Colchicine and NSAIDs should not be used for patients with renal failure, who are usually given intra-articular or systemic steroids. Further, with hypertension and type 2 diabetes mellitus, steroids can be poorly tolerated, especially when repeated courses are needed in patients with frequent flares. Following the observation that interleukin 1 (IL-1) plays a major role in MSU crystal-triggered inflammation [106], IL-1 blockers have been proposed for the management of flares for patients with difficult-to-treat disease. Open studies of the IL-1 receptor antagonist anakinra [107, 108] support its off-label use for patients resistant or with contraindications to NSAIDs, colchicine and steroids. Canakimumab, a long-lasting antibody to IL-1β, has been approved by the European Medical Agency (but not the US Food and drug Administration), following two RCTs comparing the intra-muscular triamcinolone acetonide [109]. The EULAR has recommended considering IL-1 blockers for the management of gout flares in patients with frequent flares and with a contraindication to NSAIDs, colchicine and steroids (oral or injectable) [94]. Current infection is a contraindication.

According to the EULAR and American College of Rheumatology (ACR) recommendations [94, 110], small doses of colchicine should be used during the first 6 months of ULD therapy to decrease the risk of ULD flares. Colchicine exposure has been found to be up to two-fold higher in people with moderate or severe renal impairment [111], thereby exposing CKD patients with chronic prescription of small-dose colchicine to increased risk of toxicity [112]. A (reversible) neuromuscular toxicity has been described in CKD patients [113]. Therefore, the colchicine dose should be reduced in CKD patients. Co-prescription of statins [114] or drugs interacting with colchicine by inhibiting cytochrome P450 or 3A4**/**P-glycoprotein [115] should be avoided in patients with renal failure.

### Impact of comorbidities on urate-lowering treatment

Comorbidities also interfere with the use of ULD to treat gout. The increased risk of premature death and the increasing prevalence of comorbidities with disease duration [6] have contributed to the recommendation by the EULAR to consider ULD indication early after a definite diagnosis of gout [94]. According to the EULAR and ACR, CKD should prompt the indication for ULDs [94, 110].

Patients with heart failure are often prescribed furosemide, which has been found to decrease the hypouricaemic effect of allopurinol while increasing the blood concentration of oxypurinol [116]. This paradox appears to rely on a recently described additional mode of action of allopurinol, reducing XO expression in vitro, a mechanism that is abolished by furosemide [117]. Cardiovascular diseases, and not diuretics, might increase the rate of serious cutaneous adverse reactions (SCARs) to allopurinol. Indeed, a recent nationwide study in Taiwan found that cardiovascular (and renal) diseases increased the risk of allopurinol hypersensitivity, with no association seen with diuretic use [118].

The dose of allopurinol for CKD patients is debated between European and US gout experts [119, 120]. Most country agencies have reduced the maximum approved dose of allopurinol according to creatinine clearance following a report of high doses of allopurinol being associated with SCARs in CKD patients [121]. This dose reduction translates into a frequent inability to reach the desired uricaemia [122]. When designing their recommendations, ACR experts were confident in the risk minimisation obtained with slow increases of allopurinol dosage as observed in a retrospective case–control study [123] and recommended titrating allopurinol above the authorised dose, if necessary, to reach the uricaemia target [110]. The EULAR committee had a more conservative approach, taking into account the rarity of SCARs [124], which leads to great uncertainties in defining risk factors, a recent report of increased severity of skin reactions in CKD patients with sustained oxypurinol levels [125], and the availability of alternative drugs such as febuxostat, which was approved without dosing changes for mild and moderate renal failure. The EULAR recommendations retained the limitation of the maximum daily dose in CKD patients according to creatinine clearance. If the limited dose did not allow reaching of the target, the EULAR recommended a shift to alternative drugs such as febuxostat.

## Conclusion

Renal and cardiovascular comorbidities are frequent in patients with gout and play an important role in the premature mortality observed in the disease. Patients with gout should therefore be systematically screened for renovascular diseases and risk factors, which should be addressed as an important part of gout management. Comorbidities, especially renal disease, and drugs prescribed for their management should be considered during the choice of drugs to be used in the treatment of gouty inflammation and for urate lowering. Finally, whereas an impressive number of epidemiological data have shown the association of renal and cardiovascular diseases with asymptomatic hyperuricaemia, causality remains uncertain, and large RCTs are still needed to assess the cardiovascular and renal benefits of asymptomatic hyperuricaemia treatment.
